# Evolution of biological sequences implies an extreme value distribution of type I for both global and local pairwise alignment scores

**DOI:** 10.1186/1471-2105-9-332

**Published:** 2008-08-07

**Authors:** Olivier Bastien, Eric Maréchal

**Affiliations:** 1 UMR 5168 CNRS-CEA-INRA-Université J. Fourier, Laboratoire de Physiologie Cellulaire Végétale; Département Réponse et Dynamique Cellulaire; CEA Grenoble, 17 rue des Martyrs, F-38054, Grenoble cedex 09, France

## Abstract

**Background:**

Confidence in pairwise alignments of biological sequences, obtained by various methods such as Blast or Smith-Waterman, is critical for automatic analyses of genomic data. Two statistical models have been proposed. In the asymptotic limit of long sequences, the Karlin-Altschul model is based on the computation of a *P-value*, assuming that the number of high scoring matching regions above a threshold is Poisson distributed. Alternatively, the Lipman-Pearson model is based on the computation of a *Z-value *from a random score distribution obtained by a Monte-Carlo simulation. *Z-values *allow the deduction of an upper bound of the *P-value *(1/*Z-value*^2^) following the TULIP theorem. Simulations of *Z*-*value *distribution is known to fit with a Gumbel law. This remarkable property was not demonstrated and had no obvious biological support.

**Results:**

We built a model of evolution of sequences based on aging, as meant in Reliability Theory, using the fact that the amount of information shared between an initial sequence and the sequences in its lineage (*i.e.*, mutual information in Information Theory) is a decreasing function of time. This quantity is simply measured by a sequence alignment score. In systems aging, the failure rate is related to the systems longevity. The system can be a machine with structured components, or a living entity or population. "Reliability" refers to the ability to operate properly according to a standard. Here, the "reliability" of a sequence refers to the ability to conserve a sufficient functional level at the folded and maturated protein level (positive selection pressure). Homologous sequences were considered as systems 1) having a high redundancy of information reflected by the magnitude of their alignment scores, 2) which components are the amino acids that can independently be damaged by random DNA mutations. From these assumptions, we deduced that information shared at each amino acid position evolved with a constant rate, corresponding to the information hazard rate, and that pairwise sequence alignment scores should follow a Gumbel distribution, which parameters could find some theoretical rationale. In particular, one parameter corresponds to the information hazard rate.

**Conclusion:**

Extreme value distribution of alignment scores, assessed from high scoring segments pairs following the Karlin-Altschul model, can also be deduced from the Reliability Theory applied to molecular sequences. It reflects the redundancy of information between homologous sequences, under functional conservative pressure. This model also provides a link between concepts of biological sequence analysis and of systems biology.

## Background

Automatic analysis of biological sequences is crucial for the treatment of massive genomic outputs. Our understanding of more than 90 % of protein sequences stored in public databases, deduced from automatic translation of gene sequences, will not result from direct experimentation, but from our ability to predict informative features using *in silico *workflows [1,2]. An underlying postulate is that the molecular sequences determined in biological individuals or species, which have evolved from a common ancestor sequence and are therefore homologous, have conserved enough of the original features to be similar. Popular sequence alignment methods, such as Blast [3] or Smith-Waterman [4] algorithms are used as a starting point for homology searches. All these methods computes a score *s*(*a*, *b*) between two sequences *a *and *b*. They use scoring matrices to maximize the summed scores of compared residues and find optimal local alignments, computed with a dynamic programming procedure [3,4]. Scoring matrices have been found to be similarity matrices as well [5]. Many similarity matrices are available [6-8] and evaluation studies led to the conclusion that all can be considered as log-odds ratio matrices, including the BLOSUM family [7] and the PAM family [6]. Log-odds ratio matrices are defined by $s(i,j)=\log(\frac{\omega (i,j)}{\nu (i)\nu (j)})$ where *ω*(*i*, *j*) is the joint probability of the amino acid pair (*i*, *j*), and *ν*(*i*) and *ν*(*j*) the probabilities of the amino acids *i *and *j *in the two aligned sequences.

Because re-examination of alignments obtained after massive comparisons is not manageable, confidence in alignment score probabilities is critical for automatic sequence comparisons, clustering of orthologs and paralogs, homology-based annotations or phylogeny reconstructions based on pairwise alignments [2]. Assessing whether a computed alignment is evolutionarily relevant or whether it could have arisen simply by chance is therefore a question that has been extensively studied (for review: [9]). Two major methods have been proposed.

The first and oldest method, proposed by Lipman and Pearson [10] and described extensively by Comet et al. [11] and others [12-14], uses Monte Carlo simulations to investigate the significance of a score, *s *calculated from the alignment of two real sequences *a *and *b*. This method consists in computing *η *alignments of *a *with sequences obtained after shuffling *b *[15]. The random sequence corresponding to the shuffled sequence *b *is termed *B*. The *η *alignments allow an estimate of an empirical mean score ($\hat{\mu }$) and standard deviation ($\hat{\sigma }$) from the distribution of the random variable *S*(*a*, *B*). A *Z*-*value *is then defined as:

(1) $$Z(a,b^{*})=\frac{s-\hat{\mu }}{\hat{\sigma }}$$

where * indicates the sequence that was submitted to randomization.

In practice, the computation of *Z*(*a*, b*) is known to be convergent and depends on the accuracy of the estimation of *μ *and *σ*, and therefore on *η*, ranging usually from 100 to 1000 [11,16]. Bacro and Comet [12] showed that the asymptotic law of the *Z-value *(when *η *→ ∞) was independent of the length and composition of sequences. Bastien et al. [13] further demonstrated that regardless of the distribution of the random variable *S*(*a*, *B*), the relation

(2) $$P(S(a,B))\leq \frac{1}{Z{\left(a,b^{*}\right)}^{2}}$$

is true. This relation, known as the TULIP theorem, shows that the *Z*-*value *computed for pairwise sequence alignments 1) provides an upper bound of alignment score probability [13], 2) can be used to reconstruct molecular phylogenies [14] and 3) is an accurate clustering criterion to reduce the diversity of protein sequence databases [17]. Here we call *T-value *the upper bound deduced from the TULIP theorem, *i.e. *1/*Z*(*a*, b*)^2^.

Simulations of *Z*-*value *distribution [11,18] shows that it fits a Gumbel distribution, suggesting that the distribution of alignment scores might follow a Gumbel distribution as well [19].

The second and most popular method proposed by Karlin and Altschul [20] is an estimate of the probability of an observed local ungapped alignment score according to an extreme value distribution (or EVD; for review: [19]), *i.e. *a Gumbel distribution, in the asymptotic limit of long sequences. The remarkable Karlin-Altschul formula is the consequence of interpreting the number of highest scoring matching regions above a threshold by a Poisson distribution. Briefly, considering *A *and *B *two random sequences, *m *and *n *their lengths, given the distribution of individual residues (*i.e. *amino acids), and given a scoring matrix, the number of distinct local alignments with score values of at least *s *is approximately Poisson distributed with mean

(3) *E*(*s*) ≈ *K*.*m*.*n*.exp(-*λ*.*s*)

where *λ *and *K *can be calculated from the scoring matrix and average sequence compositions based on the Poisson distribution hypothesis. *E*(*s*) is known as the *E-value*. As a consequence, if *s *is the score obtained after aligning two real sequences *a *and *b *(with *m *and *n *their respective lengths), the probability of finding an ungapped segment pair with a score lower than or equal to *s*, follows a Gumbel distribution:

(4) *P*(*S*(*A*, *B*) ≤ *s*) ≈ exp(-*K*.*m*.*n*.exp(-*λ*.*s*))

where *S*(*A*, *B*) is the random variable corresponding to the score of two random sequences. The *P-value*, defined as the probability of finding an ungapped segment pair with a score higher than *s*, is simply given by 1-*P*(S(*A*, *B*) ≤ *s*). Using the Taylor Expansion of equation (4), the *P-value *is approximated by the *E-value *when *E*(*s*) < 0.01. The validity of the Karlin-Altschul model depends on restrictive conditions: firstly, the residue distributions in the compared sequences should not be "too dissimilar" and secondly, the sequence lengths (*m *an *n*) should "grow at roughly equal rates" [20]. The length dependency of alignment scores has been discussed [20,21]. In particular, it has been demonstrated that the growth of the best matching score of gapped alignments was linear when gap penalties were small, becoming logarithmic when increasing sequence length and for larger gap penalties [21]. Although the Karlin-Altschul formula given by equation (4) is not valid for gapped alignments and although no asymptotic score distribution has been analytically established for local alignments allowing gaps, simulations [11,18,22,23] showed that, for both local and global alignments, the Gumbel law was well-suited to the distribution of scores after pragmatic estimation of the *λ *and *K *parameters.

Noticeably, this model relies on the fact that *λ *is the unique positive solution to the equation $\sum_{i,j}\nu_{a}(i)\nu_{b}(j)\exp\{\lambda .s(i,j)\}=0$, for the 20 × 20 combinations of *i *and *j *amino acids, with *ν*_*a*_(*i*) and *ν*_*b*_(*j*) the probabilities of amino acids *i *and *j *in sequences *a *and *b *respectively and *s*(*i*, *j*) the score in the substitution matrix. From a theoretic point of view, and regardless of the practical performance of the Karlin and Altschul [20] model, the fact that an observed distribution (the distribution of scores of real compared sequences) depends on a presupposed and pre-calculated parameter is not satisfactory. It would be more satisfactory if *λ *arose as a property of a biological process and/or features. We addressed therefore the question of the missing biological rationale to parameters, particularly *λ *and *K*, that proved to be valid in pragmatic terms.

In this paper, we deduced biological rationale for the Gumbel-like distribution of sequence alignment scores and *Z-values*, based on a limited number of assumptions on sequences evolution. An ancestral sequence is the origin of a lineage of homologous sequences that are subjected to evolutionary mechanisms. We considered homologous sequences as entities sharing structural features, in particular some conserved or functionally similar amino acids detected by alignment methods. Features that are preserved in two homologous sequences are estimated by a shared amount of information (SAI). In this model, the amount of information shared between an initial sequence and the sequences in its lineage (*i.e.*, mutual information in Information Theory) is a decreasing function of time: over time, some substitutions of amino acids by others having redundant properties (SAI at the residue level) may be permitted without functional break down, but leads to a decrease of the SAI between the sequences. Classically, molecular evolution is formalized with Markovian models for residue substitutions, allowing the backward reconstruction of sequences' evolution with the assumption that the proteins have been selected for a functional conservation. Here, proteins were considered as systems, with a high level of structural redundancy, which components may "age" over evolution, and "die" in case of loss of the initial amount of information required to operate accurately for a given biological function. Assumptions are therefore generalist regarding the process of sequence evolution, should it be strictly Markovian or not, but they give a formalism to the reliability of the sequences reflecting the functional status of the folded and maturated protein, and being a criterion on which positive selection pressure might act. We introduced therefore principles of the *reliability theory of aging and longevity *[24], that apply to a wide range of other systems, from artificial machines to biological population or organisms, applied here to molecular sequences. Based on the deduced model, we could provide biological basis for the *Z-value *Gumbel distribution, and significance for the corresponding Gumbel parameters (termed *K' *and *λ'*). Moreover, the assumption that the score between two sequences *a *and *b *should be the highest possible score between *a *and *b *is not necessary to observe an extreme values distribution for sequence alignment scores.

Major points of the following demonstration are:

i. The evolution of biological sequence is formalized by the evolution of the SAI between an initial sequence and sequences of its lineage. It is known that for two sequences *a *and *b*, this is measured by the mutual information I(*a*; *b*), based on Information Theory and is exactly the score s(*a*, *b*) computed with standard methods in sequences comparisons [14].

ii. If a sequence evolves, the probability that it stays near its "last" position in the sequence space is low and the longest the sequence, the lowest this probability (consequence of the concentration in a high dimensionality space [25]). The amount of information shared between an initial sequence and the sequences in its lineage decreases with time: as a consequence, one can indifferently use I(*a*; *b*) as a measure of the divergence time.

## Results and discussion

### Assumptions for a model of sequences' evolution

A basic process in the evolution of proteins is the change of amino acids over time. In the simplest view, these changes lead to amino acid substitutions, insertions or deletions. Dayhoff et al. [6] introduced the description of this process as a continuous-time Markov chain with a matrix of transition probabilities for the substitutions of any amino acid into another through time. This model allows forward and backward expressions of sequence evolution, under time homogeneity assumption, and is therefore an important tool for phylogeny reconstructions. Given a transition matrix and an equilibrium distribution of amino acids, then a matrix of amino acid substitution scores, in the sense of sequences' comparison, can be deduced [26,27].

In the generalist model described here, assumptions regarding the process of sequence evolution were not formalized, should this process be strictly Markovian or not. Given two sequences, one can, one the one hand, compute a score using dynamic algorithms [3,4] and deduce the distribution of random scores from transition matrices under the hypothesis that the two sequences have evolved according to a continuous-time Markov chains process. On the other hand, Henikoff et al. [7] demonstrated the possibility to calculate efficient log-odd matrices without the need of this assumption. Altschul [28] and Bastien et al. [14] demonstrated that log-odd matrices could be reformulated in the Information Theory framework. In particular, a score between two amino acids *i *and *j *can be interpreted as the mutual information between these two residues. At the 3D folded protein level, a molecular function emerges from the information encrypted in the amino acid sequence, and positive selection pressure acts therefore at the sequence level, maintaining a sufficient portion of the initial information, and consequently the functional status of the folded and maturated protein. We therefore focused on the evolution of the information shared between an initial sequence and the sequences of its lineage through time.

### Reliability theory and biological sequences evolution

The Reliability Theory is a general theory about systems aging, in which the failure rate (the rate by which systems deteriorate) is related to the systems longevity (For review, [24]). The system can be a machine with structured components, or a living entity or population. "Reliability" of a system (or of one of its components) refers to its ability to operate properly according to a standard [29]. The relation between the age of a system and its failure rate shows that aging is a direct consequence of redundancies within the system. For instance, when applied to a biological system in which redundant vital structures ensure a function, damage of a component that is compensated by another redundant intact one, does not lead to a complete impairment of the system. Defects do accumulate, resulting in redundancy exhaustion and giving rise to the phenomenon of aging. As the system (or one of its components) degenerates into a system with no redundancy, new defects can eventually lead to death. Reliability of the system (or component) is described by the "reliability function" *R*(*x*), also named "survival function", which is the probability that the system (or component) will carry out its mission through time *x *[30], expressed as the probability that the failure time *X *is beyond time *x*:

(5) *R*(*x*) = *P*(*X *> *x*) = 1 - *P*(*X *≤ *x*) = 1 - *F*(*x*)

where *F*(*x*) = *P*(*X *≤ *x*) is a cumulative distribution function [24] reflecting the resistance of the system to failures (at time *x*, distribution of the probability that the system could have failed previously). *R*(*x*) evaluates therefore the probability that the systems becomes completely defective after a time *x *(*x *can be a direct measure of time *t *or an increasing function of time).

The "hazard rate"*h*(*x*), also called "failure rate", is defined as the relative rate for reliability function decline:

(6) $$h(x)=-\frac{dR(x)}{R(x).dx}=-\frac{d(\log R(x))}{dx}$$

Hazard rate is equivalent to mortality force in demography [31,32]. When *h*(*x*) is a constant *h*, the system does not deteriorate more often with age, and is therefore a *non-aging *system. In this case, a simple integration of equation (6) leads to

(7) *R*(*x*) = *R*(0)exp(-*h*.*x*)

which is the exponential distribution that characterizes non-aging systems. Interestingly, a system with redundant *non-aging components *can be an *aging system*. That is to say the hazard rate of a system of components depends can depends of time whereas the hazard rate of components do not

As discussed by Gavrilov and Gravrilova [24], the "reliability theory" provided explanations for some fundamental problems regarding aging, longevity, death of organisms within populations. Organisms or populations are considered as systems in which categories of components (molecules, biological processes, cells, individuals, etc.) can be highly redundant, and be key elements for the system longevity.

Here, we propose to consider the particular case of *protein sequences as a system*, in which redundancy is ensured:

i. by the number of residue positions involved in the evolution process.

ii. at the residue level by the existence of functionally redundant amino acids (*e.g. *after a DNA damage that leads to a genetic mutation, an aspartic acid may be substituted by a functionally redundant glutamic acid), *i.e. *the existence of a SAI for all amino acids pairs.

In this model, evolutionary time is negatively correlated to the amount of information shared between an initial sequence and sequences in its lineage (SAI decreases with time, see below).

### The conservation rate: a mathematical tool to study the evolution of the information shared by biological sequences

To measure the rate of conservation of a shared structure/function relationship at time *x *within a system of homologous proteins (*i.e. *the time of observation), we considered that the decay of information shared between an original sequence and sequences of its lineage was a function of time, and therefore a mean to measure time. Evolutionary time is therefore measured here in information units. We defined an *information conservation rate *Ψ as follows:

#### Definition

Given the cumulative distribution function *F*(*x*) = *P*(*X *≤ *x*) (Probability that the system shared less than *x *information units with a reference), supposed continuously differentiable, the *conservation rate *Ψ is given by:

(8) $$\psi (x)=\underset{dx\to 0}{\lim}\frac{P(x-dx<X\leq x/X\leq x)}{dx}$$

The *conservation rate *is simply related to the hazard function, measuring a quantity that decreases over time (shared information) instead of a quantity that increases over time (age). Given *f*(*x*) = *dF*(*x*)/*dx *the density function of *x*, this conservation rate has the following properties.

(9) $$\psi (x)=\frac{f(x)}{F(x)}=\frac{f(x)}{P(X\leq x)}$$

and as corollaries:

(10) $$\psi (x)=\frac{d(\log F(x))}{dx}$$

(11) $$P(X\leq x)=\exp(-\int_{x}^{+\infty }\psi (u)du)$$

(12) $$f(x)=\psi (x)\exp(-\int_{x}^{+\infty }\psi (u)du)$$

### Derivation of the distribution of sequence alignment scores based on the distribution of mutual information between amino acids

Dobzhansky [33] and Wu et al. [34] established that *information *harbored by a protein 1) emerged from the three-dimensional self organization of its residues (*i.e. *the sequence of amino acids) and had to do with information harbored by amino acids, and 2) was submitted through time to evolutionary pressure (achievement of a minimal functional level fitting environmental and species survival conditions). Using previous empirical results [6,7,35], Bastien et al. [14] have shown that the alignment score of two homologous sequences *a *and *b *was proportional to the estimate of the SAI due to their common origin and parallel evolution under similar conservative pressure, *i.e. *the *mutual information I*(*a*; *b*) between the two events *a *and *b *in the sense of Hartley [36,37]:

(13) *s*(*a*, *b*) = *ξ*.*I *(*a*; *b*)

with *ξ *a constant defining the unity (*ξ *= 1, in bits) and *s*(*a*, *b*) the sum of the elementary scores for all aligned positions (including gap opening and gap extension penalties). Mutual information between two events *a *and *b *(differing from the mutual information defined between random variables, see [14,38]) measures the information gained by the knowledge of event *a *on the occurrence of event *b*. The mutual information being additive, *I*(*a*; *b*) is the sum of the mutual information of aligned residues, *reflecting the magnitude of the redundancy between the sequences at the amino acid level*. Mutual information between residues is therefore simply deduced from the 20 × 20 amino acid substitution matrix [6-8,35] used to compute the alignment.

Inside a given sequence, mutual information was also shown to *reflect the dependency of close or remote amino acids*, a phenomenon known as the residue co-evolution, due to their co-contribution to the sequence function [39,40].

Considering a *protein *as a *system*, which *components *are *amino acids*, we examined the mutual information between the original components and their descendants, and how amino acid mutation affected the evolution of mutual information between proteins. We simply hypothesized that an amino acid may mutate over time following random DNA mutations and look at the behavior of the entire system, namely the protein which can be measured here by the mutual information between the initial residues and the new ones, *i.e. *the corresponding substitution scores in a 20 × 20 substitution matrix. The substitution matrix is considered as an estimate of the mutual information between residues because it was computed from real sequences' data [6-8,35].

**Figure 1:**
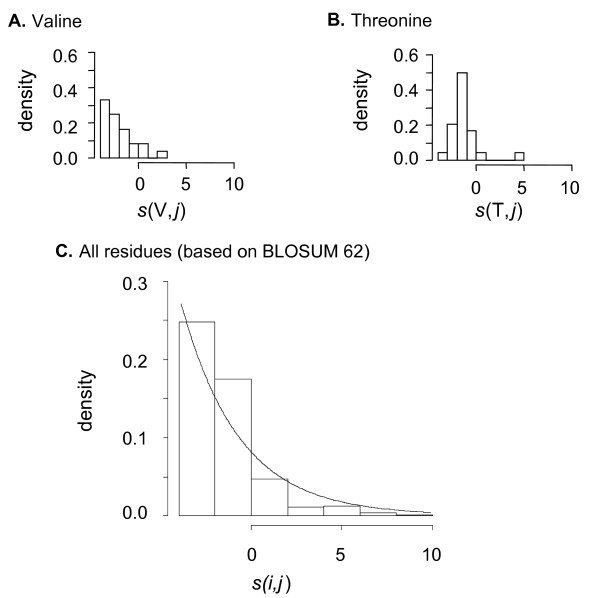
**Aging properties of amino acids**. Protein sequences are considered as systems, which components are amino acids. Over time, either amino acids are conserved (similarity of a residue with its descendant is that of identity, diagonal term of a substitution matrix) or modified due to random DNA mutations. Similarity decreases therefore with time, since no similarity is higher than that of identity. When the similarity falls below a threshold that is necessary for the residue to operate according to a standard (functional conservation), the component is damaged. **(A) Score distribution corresponding to valine substitution. **In this case, the score distribution is exponential, suggesting that valine (V) is a non-aging component. Based on BLOSUM62, residues of this type are V, F, P, W, Y, E, G, H, I, L, K, R, N, D and C **(B) Score distribution corresponding to threonine substitution. **The score distribution shows a peak, indicating a probable accelerated process of aging (functional damage) when the residue is substituted by random mutation in some other amino acids. Based on BLOSUM62, residues of this type are T, S, M, A and Q. **(C) Score distribution in the BLOSUM62 similarity matrix. **The complete distribution in the BLOSUM62 matrix is exponential (0.287.exp(-0.287.(*s*+4))), supporting a general model of amino acids as nonaging components. The exponential law for positive scores is characterized by the same parameter (*λ' *= 0.287). The original residue is termed *i*; its descent is termed *j*.

Over time, an amino acid *i *is either conserved or substituted. The similarity of *i *in an initial sequence compared with residues at the same position in protein descendants is therefore either that of identity (the diagonal term in the scoring matrix) or a lower value(no score is higher than that of identity). In average, the magnitude of the similarity of *i *compared with its descendants, related to mutual information following equation (13), is therefore a decreasing function of elapsed time. On a functional point of view, the probability that *i *was mutated into a residue with a score *S*_*i *_lower than a threshold *s*_*i *_defined to allow the component to operate like *i*, can be deduced from the distribution of substitution scores. For most amino acids (F, P, W, Y, V, E, G, H, I, L, K, R, N, D and C), the distribution of scores deduced from BLOSUM 62 fits an exponential distribution (see the case of valine in Figure 1A. For five amino acids (M, S, T, A and Q), the distribution of scores does not fit an exponential distribution (see the case of Threonine in Figure 1B). Taking the average situation, the distribution of scores deduced from the BLOSUM 62 matrix is exponential-like (Figure 1C) supporting a general model for amino acids mutual information distribution: The probability *P*_*r *_that a residue *i *is mutated into a residue with mutual information below *s*_*i *_is:

(14) *P*_*r*_(*S*_*i *_≤ *s*_*i*_) = 1-exp(-*λ*_*i*_.*s*_*i*_)

where *λ*_*i *_is the *constant information hazard rate, or failure rate, for reliability function decline of the amino acid mutual information*.

**Figure 2:**
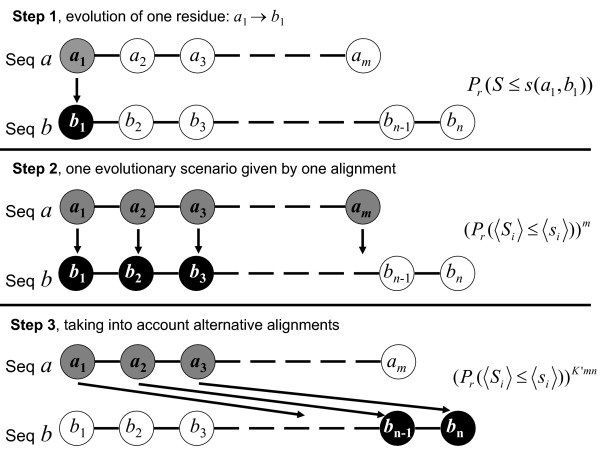
**Computing of the probability that the amount of information shared by two sequences, *S*, is lower than a threshold *s***. Given an initial sequence *a*, we can envisage different scenarios for its evolution into another sequence *b*. In a first step (**Step 1**), an elementary probability is computed by taking into account the evolution of just one residue (here *a*_1 _into *b*_1_). Considering one possible evolutionary scenario (**Step 2**), residues are considered as independent and the probability is the product of elementary probabilities for each positions aligned in this scenario, with approximations in the asymptotic limit of long sequences. The final probability (**Step 3**) is then estimated by taking into account all the possible evolutionary scenarios.

Given a sequence *a*, what is the probability that any of its *m *residues (termed *i*) had previously mutated into the *n *residues (termed *j*) of a sequence *b *and leads to the observed mutual information between sequence *a *and sequence *b*? We can consider *m *≠ *n *due to insertion or deletion events. If *m *and *n *are large, we can state the following asymptotic approximations: *S *≈ *m *⟨*S*_*i*_⟩, with $\langle S_{i}\rangle =\underset{m\to \infty }{\lim}\frac{S}{m}$ and *s *≈ *m *⟨*s*_*i*_⟩, with $\langle s_{i}\rangle =\underset{m\to +\infty }{\lim}\frac{s}{m}$ where *s *(respectively *S*) is the score between the sequence *a *(respectively *A*) and the sequence *b *(respectively *B*) (for discussion of these approximations, see [41]). In the asymptotic limit of long sequences, we can envisage different scenarios for the evolution of *a *into *b*:

In a first step (Figure 2, step 1), the probability that one residue *a*_1 _is mutated into a residue *b*_1 _with mutual information below *s*_*i *_is given:

(15) *P*_*r*_(*S*_*i *_≤ *s*_*i*_) = *P*_*r*_(*S*_*i *_≤ *s*(*a*_1_,*b*_1_)) ≈ *P*_*r*_(⟨*S*_*i*_⟩ ≤ ⟨*s*_*i*_⟩)

Considering one possible evolutionary scenario, *i.e. *one alignment (Figure 2, step 2), residues are considered as independent and the probability is the product of elementary probabilities for each positions aligned in this scenario. For the alignment of the *m *amino acids of sequence *a*, we obtain the following probability:

(16) *P*_*scenario*1 _(*S *≤ *s*) = (*P*_*r*_(⟨*S*_*i*_⟩ ≤ ⟨*s*_*i*_⟩))^*m*^

Alternative scenarios are also possible (Figure 2, step 3). The final probability is therefore computed taking into account all possible evolutionary paths (all possible alignments, Figure 2, step 3) and using *K'*<1 a correcting factor for edge effects, deletion and insertion points:

(17) *P*(*S *≤ *s*) = (*P*_*r*_(⟨*S*_*i*_⟩ ≤ ⟨*s*_*i*_⟩))^*K*'*mn*^

Considering the approximation of ⟨*S*_*i*_⟩ and ⟨*s*_*i*_⟩ respectively by *S*/*m *and *s*/*m*, we deduce the final formula:

(18) *P*(*S *≤ *s*) = (*P*_*r*_(*S *≤ *s*))^*K*'.*m*.*n*^

The density function *f*(*s*) is therefore given by:

(19) $$f(s)=\frac{dP(S\leq s)}{ds}=K^{\prime }.m.n.f_{r}(s).{\left(P_{r}(S\leq s)\right)}^{K^{\prime }.m.n-1}$$

with $f_{r}(s)=\frac{dP_{r}}{ds}(s)$ the density of the probability *P*_*r*_(*S *≤ *s*) that a residue is mutated into another

with mutual information below *s*. We can then deduce the *homology longevity rate *Ψ, defined earlier as a function of the pairwise alignment score:

(20) $$\psi (s)=\frac{f(s)}{P(S\leq s)}=\frac{K^{\prime }.m.n.f_{r}(s).{\left(P_{r}(S\leq s)\right)}^{K^{\prime }.m.n-1}}{{\left(P_{r}(S\leq s)\right)}^{K^{\prime }.m.n}}$$

Using the expression of *P*_*i*_(*S*_*i *_≤ *s*_*i*_) given by Equation (14) implies that:

(21) $$\psi (s)=\frac{K^{\prime }.m.n.\lambda^{\prime }.\exp(-\lambda^{\prime }.s).{\left(1-\exp(-\lambda^{\prime }.s)\right)}^{K^{\prime }.m.n-1}}{{\left(1-\exp(-\lambda^{\prime }.s)\right)}^{K^{\prime }.m.n}}=\frac{K^{\prime }.m.n.\lambda^{\prime }.\exp(-\lambda^{\prime }.s)}{1-\exp(-\lambda^{\prime }.s)}$$

Asymptotically, the information conservation rate is therefore given by

(22) *ψ*(*s*) = *K'*.*m*.*n*.*λ'*.exp(-*λ'*.*s*)

Using equation (12), we deduce that the distribution of alignment scores should respect the general form of the Karlin-Altschul formula:

(23) *P*(*S *≤* s*) ≈ exp(*K*'.*m*.*n*.exp(-*λ'*.*s*))

### Applications and Conclusion

We built a model of evolution of the information shared between an initial molecular sequence and the sequences of its lineage (*i.e. *homologous sequences). Sequences were considered as systems, which components are the amino acids that can independently be damaged by random DNA mutations. Residues harbor a functional redundancy reflected by the amino acid substitution scores.

From these assumptions, we deduced that the pairwise sequence alignment score should follow a Gumbel distribution (equation (22)). The *λ' *parameter is the information hazard rate for the reliability of amino acids' mutual information: it depends 1) on the distribution of the amino acids and 2) on the distribution of amino acid similarities deduced from a substitution matrix. The *K*' parameter has a more complex meaning, because it depends on likelihood of an alignment of two sequences, with edge effects, gaps, length difference and repartition of the information (the local score) in the alignment. It reflects therefore internal structural constraints on the evolution of sequences.

The Gumbel parameters for score alignments can be estimated by two kinds of simulations. First is by adjusting EVD to the simulated distribution of scores [19,22]. In that case, it is simpler to express the Gumbel law as

(24) $$P(S\leq s)\approx \exp(-\exp(-\frac{s-\theta }{\beta }))$$

with $\beta =\frac{1}{\lambda^{\prime }}$ and $\theta =\frac{1}{\lambda^{\prime }}\log(\lambda^{\prime }.K^{\prime }.m.n)$. The estimate of Gumbel parameters is achieved by determining *β *and *θ*, allowing an easy estimate of the *λ' *and *K*' parameters of equation (23). Second estimation of the Gumbel parameters is by computing the *Z-value *corresponding to the simulation of score distribution. Using the fact that for a Gumbel distribution, *μ *= *θ *+ *γβ *and $\sigma^{2}=\frac{\pi^{2}}{6}\beta^{2}$, then the *Z-value *allows a computation of the *β *and *θ *constants.

Simulations of *Z*-*value *distribution [11,18] showed that it fitted with a Gumbel law. Based on the Gumbel distribution of scores (equations (24) and (25)) and by an appropriate change of variable with equation (1), then the distribution of *Z*-*values *should respect the following equality:

(25) $$P(Z\leq z)=\exp(-.\exp(-z\frac{\pi }{\sqrt{6}}-\gamma ))$$

with *γ *the Euler-Mascheroni constant (*γ *≈ 0.5772). Equation (25) is the precise expression of the distribution of *Z-values *deduced by Pearson [18] from simulations. It is important to note that this expression of the *Z-value *distribution is independent of sequence lengths and amino acid distributions.

This consideration has practical implications, since it allows a refined estimate of the *P-value *based on *Z-value *computation, and a real gain over available methods, particularly in some documented cases where the Karlin-Altschul formula failed to assess the significance of an alignment. Table 1 shows for instance the different statistical estimates for the alignment of two homologous TFIIA gamma sequences from *Plasmodium falciparum *and *Arabidopsis thaliana*. The compositional bias in the proteome of *Plasmodium falciparum*, the malarial parasite, is known to limit the use of Karlin-Altschul statistics for pairwise comparisons with unbiased proteins such as those of *Arabidopsis thaliana *[42]. The TFIIA gamma subunit sequence of *Plasmodium *could not be deduced from BLASTP-based homology searches [43]. The Blastp apparent search failure was due to the overestimate of the *P-value *following the Karlin-Altschul formula (0.008, using unfiltered BLASTP, see Table 1). Alignment score *Z-value*, computed with either Blastp (P. Ortet, unpublished algorithm) or Smith-Waterman was above 10. The upper bound for the *P-value *based on the TULIP theorem, given by the formula *T-value *= 1/*Z-value*^2 ^[13], was therefore below 10^-2^. Eventually, the *P-value *deduced from the *Z-value *Gumbel distribution was below 10^-6 ^(see Table 1) indicating that, for both the Blastp and Smith-Waterman methods, the homology could be statistically assessed, even in the limit case of unbiased vs biased sequence comparisons. We noticed that the asymmetric DirAtPf100 matrix specified for *Plasmodium *vs. *Arabidopsis *comparisons that we developed earlier [8] allowed an additional gain in estimating this missed homology.

**Table 1:** Alignment statistics of the homologous Transcription initiation factor IIA (TFIIA) gamma chain sequences from *Plasmodium falciparum *and *Arabidopsis thaliana*.

Alignment method	Blastp	Smith-Waterman	
Substitution matrix	BLOSUM62	BLOSUM62	DirAtPf100

Statistics			
*P-value *(Karlin-Altschul)	0.008	NA	NA
*Z-value *(Pearson-Lipman)	10	11	12
*T-value *(TULIP theorem)	0.01	8.10^-3^	7.10^-3^
*P-value *(this work)	1.5.10^-6^	3.7.10^-7^	1.10^-7^

TFIIA gamma sequences from *Plasmodium *(UniProtKB Q8I4S7_PLAF7) and *Arabidopsis *(UniProtKB T2AG_ARATH) were aligned with Blastp and Smith-Waterman methods. Statistics were computed following the Karlin-Altschul model (as implemented in the Blastp algorithm) or the Lipman-Pearson *Z-value *model. The upper bound for the *P-value *based on the TULIP theorem is given following the formula: *T-value *= 1/*Z-value*^2^. The *P-value *deduced from the *Z-value *Gumbel distribution was computed following the model presented here. Substitution matrices were either BLOSUM62, or the asymmetric DirAtPf100 matrix specified for *Plasmodium vs. Arabidopsis *comparisons. NA: not applicable.

Besides a theoretical support for pragmatic observations, this report shows therefore that the alignment score Gumbel distribution is a particular and general evolutionary law for molecular sequences taken as dynamical systems. This model can be parameterized using the Karlin-Altschul or the *Z-Value *form. If Karlin-Altschul model parameters are well-estimated (using simulations for example), both forms are equivalent in practice as reported by Hulsen et al. [44]. This model shows that an extreme value distribution of alignment scores can arise not only by considering high scoring segments pairs. Indeed, derivation of a Gumbel distribution from maximum independent random variables is a well-known technique [19] and the Karlin-Altschul theorem was first demonstrated, based on this consideration [20]. We can now state that this distribution allows a different interpretation in the light of the Reliability Theory, reflecting the redundancy of information between sequences due to both the number of residues and the shared information between these residues. The model elaboration described here additionally provides a link between concepts of biological sequence analysis and the emerging field of systems biology, with a generalization of the aging concepts to all scales of the living world.

## Methods

Mathematical demonstrations are detailed in the Results section. Histograms and curves were built using the R package software (Statistics Department of the University of Auckland).

## Abbreviations

SAI: shared amount of information; TULIP: theorem of the upper limit of a score probability; EVD: extreme values distribution

## Authors' contributions

OB conceived the main theoretical model, designed and developed all demonstrations and drafted the manuscript. EM participated in the design of the study and helped to draft the manuscript. All authors read and approved the final manuscript.
